# Improving In Vitro–In Vivo Correlation (IVIVC) for Lipid-Based Formulations: Overcoming Challenges and Exploring Opportunities

**DOI:** 10.3390/pharmaceutics17101310

**Published:** 2025-10-09

**Authors:** Arnaud Bourderi-Cambon, Khaled Fadhlaoui, Ghislain Garrait, Emmanuelle Lainé, Imen Dhifallah, Manon Rossano, Philippe Caisse, Eric Beyssac

**Affiliations:** 1 Gattefossé SAS, 36 Chemin de Genas, 69804 Saint-Priest, France; abourderi-cambon@gattefosse.com (A.B.-C.); mrossano@gattefosse.com (M.R.); pcaisse@gattefosse.com (P.C.); 2 UMR 454 MEDIS UCA-INRAE, Université Clermont Auvergne, 63000 Clermont-Ferrand, France; khaled.fadhlaoui@uca.fr (K.F.); ghislain.garrait@uca.fr (G.G.); emmanuelle.laine@uca.fr (E.L.); imen.dhifallah@uca.fr (I.D.); 3 UMR 6023 Laboratoire Microorganismes: Génome et Environnement CNRS-UCA, Université Clermont Auvergne, 63000 Clermont-Ferrand, France

**Keywords:** lipid-based formulations, in vitro–in vivo correlation, in vitro dissolution, in vitro digestion, in silico, prediction tools

## Abstract

Lipid-based formulations (LBFs) play a crucial role in enhancing the oral bioavailability of poorly water-soluble drugs by leveraging lipid digestion and solubilization processes. However, developing robust in vitro–in vivo correlations (IVIVCs) for LBFs presents unique challenges due to the complex interplay of digestion, permeation, and dynamic solubilization. This article reviews the construction of IVIVC in the context of LBFs, highlighting the limitations of traditional methods and the need for tailored approaches. It examines the in vitro tools commonly employed for LBF characterization, such as USP dissolution tests, lipolysis assays, and combined models, and discusses their relevance to in vivo performance prediction. The review also explores the sources of in vivo data essential for validating IVIVC and describes the most popular in silico tools for predicting in vivo performance, focusing on lipid-based formulations. This work aims to pave the way for more effective and adaptable IVIVC methodologies for lipid-based drug delivery systems.

## 1. Introduction

**Figure 1:**
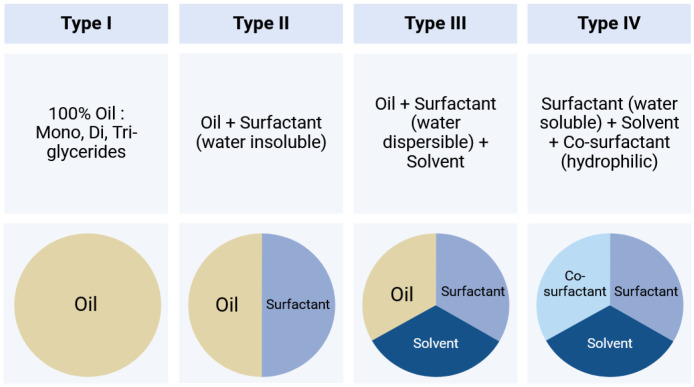
LFCS (Lipid Formulation Classification System).

Besides the chemical and physical approaches applicable to the active substance, the galenic approach is a real opportunity to improve the bioavailability of poorly soluble BCS II or IV active substances. The Biopharmaceutics Classification System (BCS) categorizes drugs based on their solubility and permeability. BCS class II compounds are poorly soluble but highly permeable, while class IV compounds exhibit both low solubility and low permeability, making them particularly challenging to develop [1]. Various approaches exist to solve this problem; salts, co-crystals, or polymorphic forms can be used to improve the solubility of the active substance. The galenic approach can also be chosen; for example, amorphous solid dispersions (ASDs), complexation with cyclodextrins, or the use of lipid-based formulations (LBFs) could enhance bioavailability. The choice of a lipid formulation is based on the use of one or more lipidic excipients mixed with the active molecule. Lipid-based formulations are used to improve the body’s exposure to active substances after oral administration. Although the main advantage of LBFs lies in the increase in apparent gastrointestinal solubility, it has also been shown that these formulations can offer advantages in terms of permeability and, in certain circumstances, promote lymphatic transport and improve bioavailability by partially avoiding hepatic first-pass metabolism [2,3,4]. LBFs can appear as Self-Emulsifying Drug Delivery Systems (SEDDSs), and depending on the droplet size formed upon dispersion, they can be further classified as SMEDDS (Self-Microemulsifying) or SNEDDS (Self-Nanoemulsifying). In the 2000s, they were classified with the aim of grouping them according to their composition. This lipid-based formulation classification system (LFCS) was introduced by Pouton [5] and updated a few years later to add a fourth formulation type [6]. LFCS classifies LBFs into four main types, according to the relative proportions of lipids, surfactants, solvents, and co-solvents (Figure 1). Type I formulations are the simplest, comprising active ingredients dissolved in an oily vehicle, such as triglycerides alone or mixed glycerides. Type II formulations include combinations of glycerides and lipophilic surfactants with a low lipophilic–hydrophilic balance (HLB). Type III formulations include mixtures of glyceride lipids and more hydrophilic surfactants with a higher HLB and may also include co-solvents. Finally, a type IV HLB classification was introduced later in response to the growing use of formulations that do not contain traditional lipids. Type IV formulations include only a combination of surfactants and co-solvents without hydrophobic compounds [6]. Beyond SEDDS and other liquid lipid-based formulations, Solid Lipid Nanoparticles (SLNs) and Nanostructured Lipid Carriers (NLCs) represent additional lipid-based delivery systems [7]. SLNs are produced by replacing the liquid lipid of an emulsion with a solid lipid or a blend of solid lipids, resulting in solid lipids dispersed in an aqueous medium and stabilized with surfactants. NLCs were developed to overcome some limitations of SLNs, as they combine solid and liquid lipids as primary structural components. This allows NLCs to achieve higher drug-loading capacity and increase stability during storage.

Despite evidence of improved oral absorption and bioavailability of poorly water-soluble compounds via LBFs, their formulation design remains complex. Unlike conventional formulations, in the case of LBFs, the behavior of lipids in the gastrointestinal tract, and, in particular, their digestion after administration, must also be taken into account. Under these conditions, there are complex interactions between physiological and physical processes, making it difficult to rationally select an optimal composition of lipid excipients [8]. Moreover, in vitro studies cannot fully mimic these processes, and the predictability of available in vitro tests can sometimes be questioned. Indeed, it is difficult to accurately predict the performance of different lipid formulations. This lack of predictability can lead to difficulties when establishing in vitro/in vivo correlations (IVIVC) in the case of LBFs [9]. Those correlations are commonly used for formulations such as controlled-release systems, where the relationship between in vitro dissolution and in vivo drug absorption is more straightforward. In these cases, dissolution is the limiting factor, which means that the prediction of in vivo performance is directly linked to the prediction of dissolution kinetics. However, for complex formulations like LBFs, the establishment of a reliable IVIVC becomes more challenging. LBFs involve dynamic processes that are not easily captured by traditional in vitro dissolution tests or in silico pharmacokinetic models. As a result, predicting in vivo performance from in vitro or in silico data for LBFs requires more sophisticated models and a deeper understanding of the physiological factors that influence drug absorption.

## 2. IVIVCs in the Context of Lipid-Based Formulations

In recent years, the concept and application of in vitro–in vivo correlation for pharmaceutical dosage forms has received considerable attention from the pharmaceutical industry, academia, and regulators. Correlations between in vitro and in vivo data are often used during pharmaceutical development to reduce development time and optimize formulation [10]. From a regulatory perspective, IVIVC enables dosage form optimization while minimizing the number of clinical trials in humans. In addition, they can be used to establish dissolution acceptance criteria and serve as surrogates for additional bioequivalence studies [10]. According to the United States Pharmacopeia (USP), an IVIVC is “the establishment of a rational relationship between a biological property, or a parameter derived from a biological property, produced by a test method or a parameter derived from a biological property produced by a pharmaceutical form, and a physicochemical property or characteristic of the same pharmaceutical form” [11]. For the Food and Drug Administration (FDA), an IVIVC is “a predictive mathematical model describing the relationship between an in vitro property of a dosage form and a relevant in vivo response” [12]. Typically, the in vitro property is the rate or extent of drug dissolution or release, while the in vivo response is the plasma drug concentration or amount of drug absorbed. In vitro–in vivo correlation (IVIVC) is a valuable biopharmaceutical tool used in drug development to predict in vivo drug performance.

However, IVIVC has several limitations that affect its development, accuracy, and applicability [13]. One major limitation is physiological variability; when a correlation is established using preclinical data, translation from animal to human physiology can challenge its validity. In addition, inherent variability between human gastrointestinal tracts can further complicate predictions. Pharmacokinetics also presents challenges for IVIVC, as drugs exhibiting nonlinear absorption or metabolism, significant first-pass effects, or involvement in active transport mechanisms are more difficult to correlate. Another important limitation is that IVIVC is formulation-dependent; a correlation established for one type of formulation may not necessarily be valid for another. Finally, the level of correlation is an important consideration; level A correlations are the most informative but can be difficult to establish for drugs with complex absorption mechanisms, while level B and C correlations provide less precise predictions and may not satisfy regulatory requirements without supplementary data. Nevertheless, for the purpose of supporting formulation design, level B and C correlations are often sufficient.

**Figure 2:**
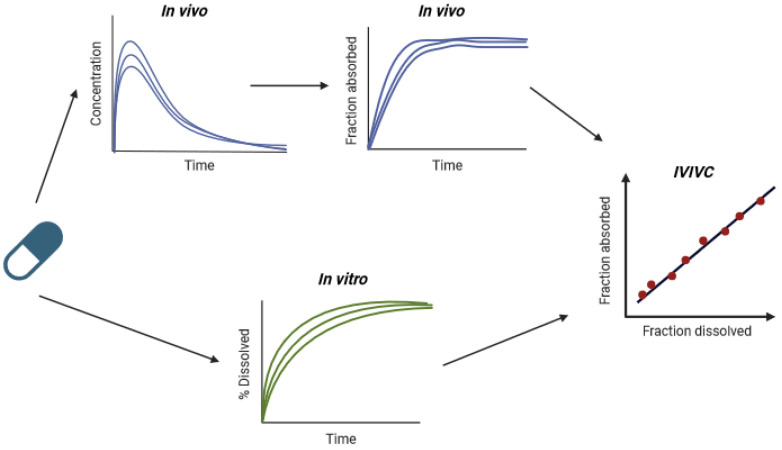
Simplified diagram of IVIVC development. The green curves represent in vitro data. The blue curves represent in vivo data.

There are different levels of in vitro–in vivo correlation, allowing relationships to be established between the dissolution properties and its in vivo performance in the case of dosage forms such as sustained release, where API release determines bioavailability [10,14]. Level A represents the most precise correlation, directly linking in vitro dissolution rate to drug entry into the body as shown in Figure 2, enabling bioavailability to be predicted without further human studies. Level B compares average dissolution and residence times in vivo, without matching plasma concentration profiles point by point. Level C links a dissolution time point to an average pharmacokinetic parameter (such as AUC or Cmax) but offers a less complete correlation. Multiple level C extends this approach to several dissolution time points, enabling certain formulation modifications to be justified. Finally, level D is a qualitative analysis or ranking with no regulatory value, mainly used to guide formulation development [10,14].

LBFs are designed to enhance the solubility and absorption of poorly water-soluble drugs, using lipid-based excipients such as oils, surfactants, and co-surfactants. However, these excipients introduce a level of complexity in the correlation between in vitro dissolution profiles and in vivo pharmacokinetics. While traditional formulations may rely on simple dissolution release testing to predict bioavailability [15], LBFs require more sophisticated models to account for the dynamics of lipid digestion, micelle formation, and lymphatic transport, for instance, which are not directly observable in basic in vitro tests. The lack of standardized, universally accepted in vitro and in silico methods that capture the full complexity of lipid-based systems can result in discrepancies between in vitro and in vivo data and between different studies.

There are notable case studies where the predicted in vivo behavior of LBFs based on in vitro data has failed to align with observed pharmacokinetic outcomes. On fenofibrate, Do et al. used in vitro dispersion data to examine the performance of four LBFs in comparison with in vivo data after administration in rats. However, the results failed to distinguish between LBFs administered in the fasted or fed state, and no correlation could be identified [16]. As examined by Feeney et al., it was found that of eight drugs studied using the pH-stat lipolysis device, only half correlated well with in vivo data [17]. Furthermore, in a paper published in 2014, Thomas and colleagues highlighted the lack of predictability of lipolysis through a study of fenofibrate and mini-pigs [18]. Another publication on an indirubin derivative failed to predict the in vivo performance of a lipid formulation with lipolysis data [19]. In the 2010s, attempts were made to establish IVIVCs for the BCS 2 molecule cinnarizine. Formulations were often similar between publications, and in vivo data were based on dog studies. Larsen et al. were only able to obtain a level D correlation and observed precipitation on one of the formulations during in vitro lipolysis, whereas in vivo performance was the same for all formulations [20]. Christophersen et al. were also able to obtain a qualitative correlation for the fasted state, but no quantitative correlation could be established for the fed state [21].

In terms of in vitro methods, dissolution tests are often used to perform in vitro–in vivo correlations, in particular the USP 2 apparatus. Lipolysis is useful for qualitative studies comparing formulations but is less suited to the development of IVIVC. Several articles demonstrate that the precipitation observed in vitro during lipolysis is not related to in vivo performance, and that studying the crystalline form of the precipitate provides a better understanding of the phenomena involved [22]. In most cases, lipolysis tests are designed to represent intestinal conditions only; therefore, lipolysis with a gastric step may have an impact on the predictivity of the method and may allow a closer mimicking of the biopharmaceutical process after administration [23].

Given the complexity of mimicking the in vivo behavior of lipid-based formulations using standard dissolution or lipolysis models, advanced approaches such as combined lipolysis–permeation models have been developed to provide deeper insights into their mechanisms. Indeed, when in vitro studies have failed to predict or reproduce the in vivo performance of LBFs, the lack of an in vitro absorption mechanism is often cited among the possible explanations for the lack of IVIVC [24]. These hybrid systems allow for a more comprehensive assessment by integrating key processes like lipid digestion and drug permeation, bridging gaps left by traditional methods. The following sections of this review will delve into these advanced tools and their applications in LBFs characterization.

To improve the success of IVIVC for lipid-based formulations, it is essential to understand and replicate their in vivo behavior. LBFs undergo digestion, forming mixed micelles that impact drug solubilization and absorption. Therefore, generating detailed in vitro data such as lipolysis profiles, dissolution, or permeability assays is a crucial first step. These data must then be linked to relevant in vivo studies to ensure translational value. When appropriately implemented, this approach could enhance predictive performance, making IVIVC a potentially powerful tool in LBF’s development.

## 3. Specific Considerations for LBFs

### 3.1. Dispersion/Digestion

When developing a new drug, the choice of formulation strategy is often guided by decision trees based on the physico-chemical properties of the active substance. In the case of LBFs, the behavior of lipids in the gastrointestinal tract, and most precisely their dispersion/digestion, must also be taken into account. One of the key characteristics of lipid-based formulations is their ability to solubilize active pharmaceutical ingredients through micellization. Surfactants can significantly enhance drug solubility by forming micelles, enabling solubilized concentrations that exceed the equilibrium solubility of the API alone [25]. Most of the time, above the CMC (Critical Micelle Concentration), the active molecule is directly dependent on the surfactant concentration. It has been observed that increasing surfactant concentrations can enhance the solubility of a molecule, a phenomenon reported for several different surfactants [26]. SEDDS are, therefore, mixtures of lipids and surfactants designed to enhance API solubilization. When they are dispersed in water or gastrointestinal fluids, especially those with high amounts of water-miscible cosolvents or hydrophilic surfactants, there may be a significant modification in solubilization capacity. If drug concentrations exceed the equilibrium solubilization capacity or precipitation does not occur immediately, the system is considered supersaturated [25]. This phenomenon is quantitatively described by the maximum supersaturation ratio (SR^M^), which reflects the supersaturation generated upon formulation dispersion and digestion [27]. SR^M^ is calculated as the ratio between the maximum solubilized drug concentration achievable before digestion without precipitation and the equilibrium solubility of the drug in the digested aqueous phase.

**Figure 3:**
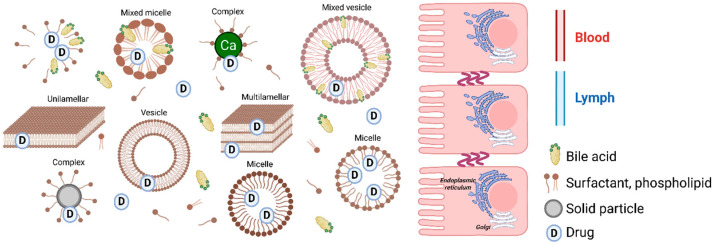
In vivo possible behavior of surfactants (adapted from Maher et al., 2023 [30]).

After oral administration of lipid formulations, components are dispersed to form lipid droplets as explained above, followed by lipolysis and solubilization of digestion products by bile acids, forming a colloidal solution of mixed micelles. During lipolysis, triglycerides are digested into diglycerides, monoglycerides, and fatty acids by lipases and co-lipases. Lipase is a digestive enzyme produced by the pancreas. It becomes active only when it encounters the surface of emulsified fat droplets, working in conjunction with bile salts and co-lipase, a co-factor found in pancreatic juice. The presence of colipase and bile salts helps the enzyme bind effectively to its substrate and promotes the emulsification of fats. Once bound, lipase breaks the ester bonds in triglycerides, resulting in the formation of free fatty acids and 2-monoacylglycerols [28]. A well-dispersed lipid formulation is, therefore, necessary to ensure a contact surface with the gastric or intestinal environment and enable homogeneous lipolysis [29]. The intercalation of these digestion products with bile secretions generates lipid reservoirs for the active substance, ranging from liquid crystalline phases at the oil/water interface and smaller multilamellar and unilamellar vesicles to mixed micellar species in the bile-rich areas of gastrointestinal fluids (Figure 3) [30]. Surfactants can adsorb to undigested solid particles, partition into and emulsify food lipids, and form colloidal structures. They can also bind to species dissolved in luminal fluid, such as proteins. These interactions reduce the amount of monomers available to interact with the membranes of the intestinal epithelium. However, in the presence of a sufficiently high concentration of surfactant, monomers can interact with the epithelium at such a concentration that membrane alterations are observed [30].

Micelles are isolated from the rest of the intestinal contents in a layer of unstirred water at the level of the intestinal mucosa and dissociated by the pH effect. The mechanism of dissociation of lipolysis products from mixed micelles is not yet fully elucidated, but it has been suggested that the process is associated with an acidic microenvironment present in the layer of unstirred water overlying the intestinal membrane. According to the microenvironment theory, ionized fatty acids are converted to their non-ionized forms and dissociate from the micelles before being rapidly absorbed across the intestinal membrane [31]. After enzymatic digestion, these lipids are taken up by enterocytes at the apical brush border by diffusion. Once absorbed, some fatty acids can move directly into the blood via diffusion, while others are recombined in the endoplasmic reticulum to form triglycerides. These triglycerides are then subsequently secreted into the circulation in the form of chylomicrons [32,33]. The particle size of the dispersion also plays an important role, with smaller colloidal structures able to diffuse more easily through the unstirred water layer and bring the active substance to the site of intestinal absorption. Given the complex interactions in the gastrointestinal tract, it seems that the properties of the dispersion formed after interaction with bile and pancreatic secretions, including particle size, condition the performance of LBFs rather than the particle size of the dispersion at the initial stage [17].

### 3.2. Absorption

**Figure 4:**
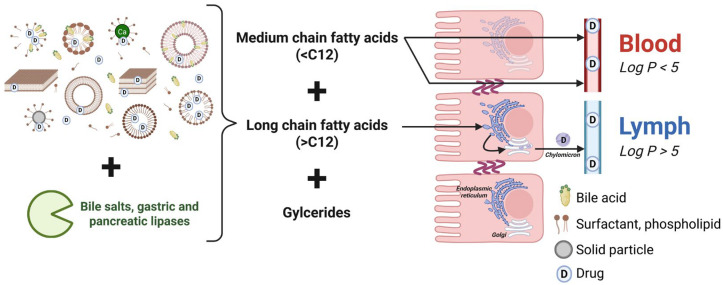
Possible effects of lipid excipients on absorption. The arrows represent the absorption pathways.

Absorption is a major factor influencing bioavailability. Absorption phenomena are described according to several modes: passive diffusion across membranes, transporter-mediated transport, endocytosis, or paracellular transport [34]. Lipid formulations have an impact on this phenomenon by promoting the passage of molecules from the intestinal lumen through the intestinal wall, with an increase in paracellular or intracellular permeability. To this end, certain lipid excipients can cause membrane fluidization, modify the type of intracellular transport, or impact the opening of tight junctions [35]. Indeed, medium-chain fatty acid esters can act as safe permeation enhancers, allowing transient and reversible opening of tight junctions between epithelial cells to promote the paracellular pathway or by interacting with the membrane to promote the transcellular pathway to the bloodstream (Figure 4) [36]. For example, the medium-chain fatty acid Labrasol^®^ ALF (caprylocaproyl polyoxyl-8 glyceride), manufactured and commercialized by Gattefossé (Saint Priest, France), was studied by McCartney et al. in 2019 [37]. Using ex vivo Ussing chamber experiments, followed by in vivo confirmation, the authors demonstrated that this excipient transiently induces mild intestinal epithelial perturbation and reversibly opens tight junctions, allowing the transport of molecules with molecular weights comparable to that of insulin. In 2024, the same research group further confirmed the absorption-enhancing properties of medium-chain fatty acids by investigating a second excipient, Labrafac™ MC60 (glycerol monocaprylocaprate) [36]. This improvement in permeability could be beneficial for BCS class III and IV active substances.

On the other hand, long-chain fatty acid esters can promote transport to the lymphatic circulation for highly lipophilic actives, thus bypassing the liver and reducing the first-pass effect and the metabolism for actives highly sensitive to this degradation pathway (Figure 4) [38,39]. This is one of the major advantages of lipid-based formulations. To achieve this, drugs are co-absorbed with long-chain fatty acids that associate with lipoproteins in chylomicrons, and the contents of these chylomicrons are then released directly into the lymphatic system [2]. Halofantrine has been used as a model drug to study lymphatic transport in the presence of lipids. By comparing short-, medium-, and long-chain fatty acid-based vehicles, the authors demonstrated that lymphatic absorption of halofantrine is significantly enhanced in formulations containing long-chain glycerides in cannulated rats [40]. Similar conclusions were observed in cannulated dogs, where 1 g of lipid-based formulations containing long-chain fatty acids also resulted in increased lymphatic uptake [41]. Although molecular weight does not appear to significantly influence lymphatic transport, the lipophilicity of a drug (expressed as log *p*) seems to impact its distribution between the blood and lymphatic system. According to several studies, lymphatic uptake from simple lipid formulations or oil solutions is minimal or absent for compounds with a log *p* < 5 [42]. Lymphatic transport is primarily evaluated using in vivo models, which remain the most established approaches. These include animals with cannulated lymphatic ducts or the administration of chylomicron secretion inhibitors such as cycloheximide [43]. However, such in vivo models are difficult to establish, and consequently, data on lymphatic transport remain limited. In contrast, in vitro methods to assess this absorption pathway are far less common. The lymphatic system is a complex biological network involving transport, resynthesis, assembly, and dissociation processes, which is difficult to replicate in vitro [32]. However, recent developments have introduced in vitro approaches based on artificial chylomicrons derived from Intralipid^®^ emulsions combined with absorption assays. These emerging models offer a preliminary evaluation of both the lymphatic transport potential of active pharmaceutical ingredients and the influence of lipid-based excipients on this process [39].

## 4. Available Data Sources for IVIVC Development

### 4.1. In Vitro Methods

**Figure 5:**
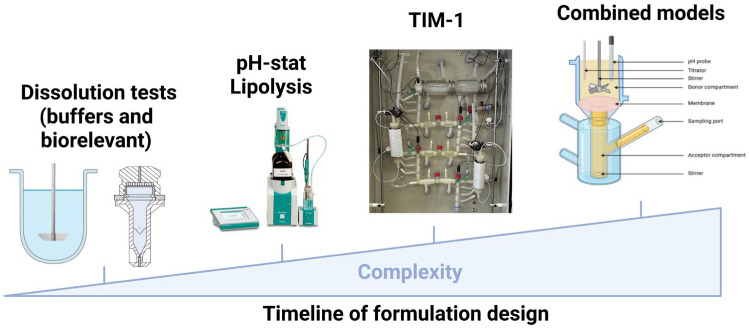
Timeline of in vitro tools applied throughout lipid-based formulation design.

Several in vitro tools are available to characterize lipid-based formulations, varying in complexity and required material and financial resources. As formulation design is a lengthy process, these tools are not always suitable at every stage of development, as shown in Figure 5. Basic dissolution tests, performed in buffer or biorelevant media, are the least complex and widely available in laboratories and pharmaceutical companies. To gain further insight into formulation behavior, lipolysis studies can be conducted to evaluate the impact of digestion on active pharmaceutical ingredient release. More complex digestion systems, such as TIM-1, and combined models incorporating an absorption step, can then be employed. These preliminary studies are subsequently followed by in vivo evaluations to identify the most promising candidates, while in silico PBPK and PBBM models provide ongoing support and guidance throughout the development process.

#### 4.1.1. Dissolution

**Figure 6:**
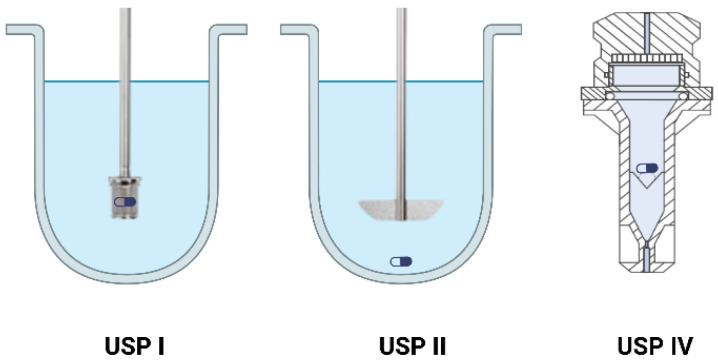
USP dissolution apparatuses.

Biopharmaceutical dissolution tests, such as those described by the United States Pharmacopeia (USP) using apparatus 1 (basket), apparatus 2 (paddle), or apparatus 4 (flow-through cell), are essential for assessing the in vitro release and dispersion of lipid-based oral formulations (Figure 6). These tests simulate the gastrointestinal environment to predict how a drug dissolves and is released from the dosage form. The equipment used for dissolution testing is numerous and well described in the literature. They each have their own characteristics, with their advantages and disadvantages [44]. These tools can be used with dissolution media such as buffers, which are characterized by a specific pH, but they can also be used with biorelevant media, which simulate parts of the gastrointestinal tract by representing fasted or fed conditions. The use of these biorelevant media in dissolution tests enables more accurate prediction of drug performance in vivo. They enable us to assess how the formulation will interact with bile salts and other components of the gastrointestinal tract, ensuring efficient drug release and absorption [45].

Fenofibrate is one of the most widely investigated molecules. Fei and colleagues have established a correlation between SMEDDS and in vivo exposure in humans. In this case, IVIVC could be obtained [46]. In addition, one study demonstrated that the use of dispersion tests provided better correlations and predictions of in vivo behavior than lipolysis tests when fenofibrate was studied in mini-pigs [47].

The most commonly used dispersion test is the USP 2 test, which has been used to correlate data generated in vitro with various in vivo data on a number of model molecules formulated with lipids: arundic acid/human [48], canaglifozin/rats [49], cyclosporin/dogs [50], dutasteride/rats [51,52], lopinavir/human [53], olaparib/rats [54], olmesartan/rats [55], ritonavir/human [56], and simvastatin/dogs [57]. In most cases cited above, level A correlations could be established; however, predictions were sometimes limited to level D [57].

Other tools can be used to carry out dispersion tests and are sometimes used to build in vitro–in vivo correlations. The USP 4 flow-through cells can be used in closed or open circuits. In 2005, a team was able to establish IVIVC under fed and fasted conditions on danazol, formulated in solution with cyclodextrin, in comparison with in vivo data on humans by using USP 4 apparatus [58]. More recently, correlations have been obtained with better accuracy for the open circuit on solid dispersions of efonidipine [59]. The use of a USP 1 basket instead of a USP 2 paddle is also one method of performing a dispersion test. Al Zahabi and colleagues constructed multiple C-level correlations with this method on lipid microparticles containing vildagliptin and in vivo data on dogs [60].

Dissolution tests are the most commonly used method to establish IVIVC [47]. However, in the case of lipid-based formulations, digestion is an important phenomenon that can cause variations in dissolution kinetics. Therefore, lipolysis tests are valuable during the pharmaceutical development of lipid-based formulations.

#### 4.1.2. Lipolysis

**Figure 7:**
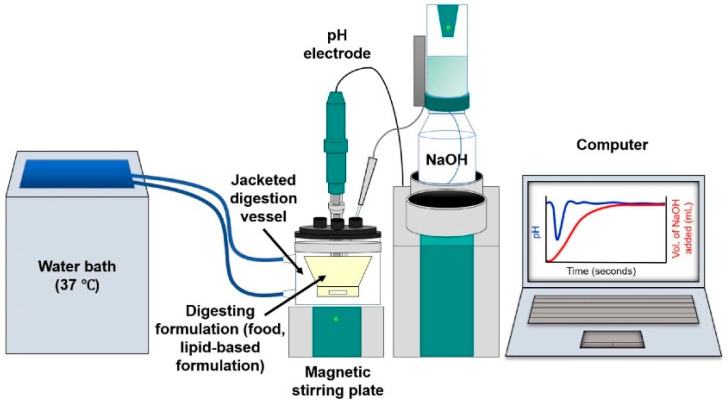
In vitro pH-stat lipolysis apparatus (from Pham et al., 2021 [62]).

The lipolysis test is used to assess lipid hydrolysis in the intestinal and gastrointestinal phases. They are essential for understanding the digestion of lipid-based oral formulations. The tools used for lipolysis experiments often include specialized equipment presented in Figure 7 called pH-stat titration systems. The test begins with a formulation dispersion step, followed by digestion of the formulation in a biorelevant medium with added enzymes [61]. Samples are taken from the digestion vessel and analyzed by liquid chromatography, usually to determine the quantity of active ingredient in solution [8]. Lipolysis can simulate intestinal or gastrointestinal conditions. Most frequently only intestinal conditions are tested, but the addition of a gastric step can highlight potential precipitation in an acidic environment and can also simulate lipid digestion, which begins in the stomach. Some lipid excipients, such as macrogolglycerides, are hydrolyzed by gastric lipase [23]. The addition of the gastric phase before the intestinal part has thus increased the predictivity of the in vivo performance of lipid formulations and the development of in vitro–in vivo correlations [23].

In most cases where lipolysis is used to guide the development of LBFs, this test is more effective in constructing level D correlations (simple qualitative comparison) than in constructing level A IVIVC [63]. Level D correlations have already been published in the literature on danazol/dogs [64,65], dexamethasone/rats [66], griseofulvin/rats [67], fenofibrate/rats [47], halofantrine/dogs [67], progesterone/rats [68], and vitamin D3/rats [68]. However, quantitative correlations are also possible. This is the case for cannabidiol, studied in 2021 by De prà and colleagues, who were able to demonstrate two IVIVCs between in vitro LBFs data and studies in mice (R^2^ = 0.75) and humans (R^2^ = 0.66) [69]. Quantitative correlations can also take the form of nonlinear correlations. McEvoy et al. constructed two nonlinear correlations on two cholesteryl ester transfer protein inhibitors: CP-529,414 torcetrapib and CP-532,623 [70].

Precipitation observed in dispersion and lipolysis tests is sometimes not representative of the in vivo performance of a lipid formulation. Since the crystallinity level or nature of the precipitate may be different from that of the native drug, a crystallographic study of the precipitated compound can help predict its redissolution and thus better anticipate the bioavailability of different formulations. The example of halofantrine was studied by Thomas and colleagues and showed that precipitation of the molecule in lipolysis tests was not transferable to in vivo performance [22].

Lipolysis tests seem, therefore, more suitable for LBFs than dispersion tests, as they simulate digestion of the formulation in the gastrointestinal tract. However, it has been noted that the in vitro data generated remain qualitative, and it is difficult to construct real in vitro–in vivo correlations. Hence, the use of fewer static tests may improve this capacity.

#### 4.1.3. Dynamic Digestion

**Figure 8:**
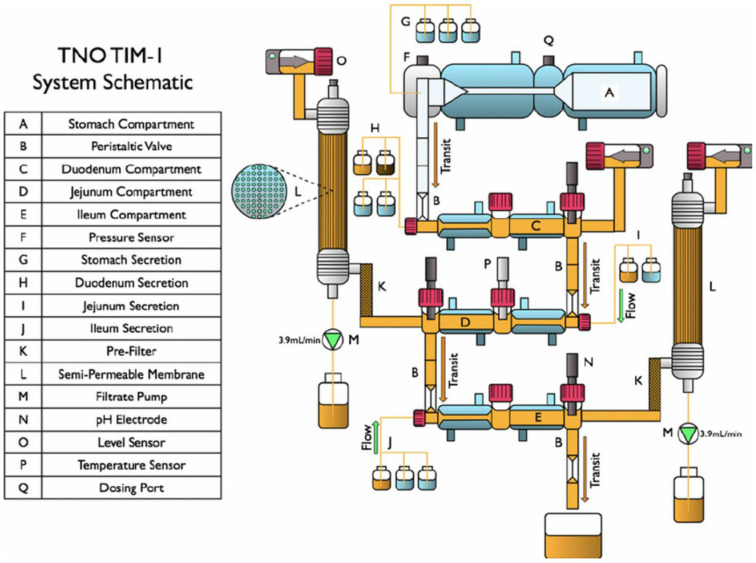
TIM-1 dynamic digestion apparatus (from Dickinson et al., 2012 [74]).

Several dynamic digestion models have been developed to simulate the complex in vivo gastrointestinal environment for studying lipid-based formulations. These models incorporate aspects of gastric and/or intestinal digestion, providing a closer approximation of physiological conditions. For example, we can mention some dynamic stomach models such as the Fed Stomach Model (FSM) [71] or the Dynamic Gastric Model (DGM) [72]. Among these, the TIM-1 (Figure 8) and tiny-TIM systems are the most widely used dynamic digestion devices. TIM-1 consists of four compartments representing the stomach, duodenum, jejunum, and ileum (see Figure 8). Each compartment is equipped with sensors and pumps to control the flow of digestive fluids and maintain appropriate pH levels. The system uses a computer-controlled mechanism to simulate the peristaltic movements and mixing actions of the gastrointestinal tract [73].

Dynamic digestion effectively predicts the influence of diet on bioavailability. In a comparison between tiny-TIM and TIM-1 on four BCS 2 and 4 model molecules (ciprofloxacin, posaconazole, nifedipine, and fenofibrate), which are not LBFs, both systems showed bioavailability data in the small intestine under fasting and feeding conditions, which were consistent with available human plasma data [75].

Data generated with TNO TIM-1 or tiny-TIM systems are also highly relevant to the development of PBPK models [76]. The first TIM-1 pharmaceutical study coupled with in silico was published by Naylor et al. [77]. In vitro TIM-1 data for paroxetine were used as input to GastroPlus^®^ (version 9.7) to predict plasma concentration. The addition of data generated with TIM-1 in a PBPK model was also useful for studying the impact of particle size and the effect of food on bioavailability [78]. Another tiny-TIM study was carried out with a BCS 2 compound. In the in silico model, data using PKPlus™ were incorporated into the GastroPlus^®^ ACAT model. Using IVIVCPlus™, the authors found an IVIVC level A [79].

Studies on lipid-based formulations using dynamic digestion tools are less frequent. In 2022, the bioavailability of ibuprofen from different formulations (tablets and soft capsules) was determined in tiny-TIM experiments with fasting and feeding conditions. The soft capsule was based on a lipid formulation of medium-chain triglycerides. The absorption of ibuprofen as a function of time was then calculated using a two-compartment in silico model. On this basis, an IVIVC was constructed, successfully predicting the behavior of different ibuprofen formulations [80]. The impact of diseases of the gastrointestinal tract was characterized with TIM-1. To this end, ciprofloxacin in oral lipid suspension was administered with different intestinal fluid compositions (bile salts, lipids) to simulate healthy and sick patients [81].

This equipment provides much more information by approximating in vivo physiological conditions than dispersion or lipolysis tests. The bioavailability of compound A6197 (BCS 1) in tiny-TIM was compared with dissolution in USP 1, 2, and 4 devices and with clinical data. Only the tiny-TIM experiments enabled the four formulations studied to be correctly classified. In vitro bioavailability data for tiny-TIM were strongly correlated with clinical values for AUC [82]. However, it is complex and costly to set up.

#### 4.1.4. Combined Models

##### Lipolysis and Permeation

**Figure 9:**
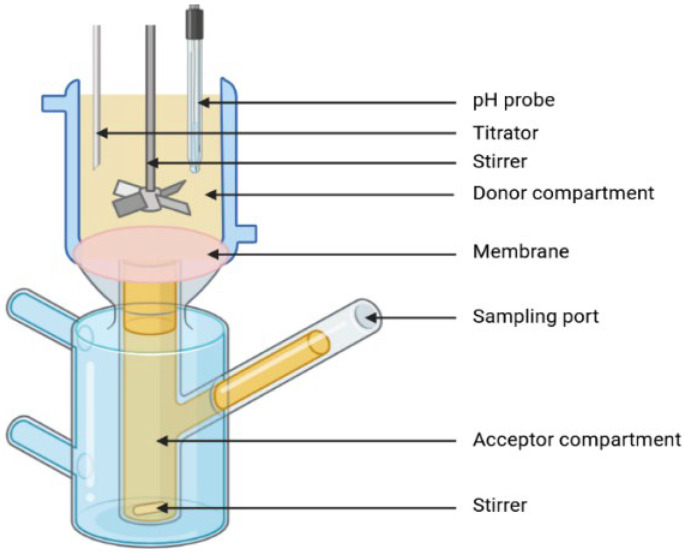
In vitro lipolysis/permeation combined model.

As mentioned above, the absence of in vitro absorption is often cited as a possible explanation for the lack of IVIVC [24]. Lipolysis–permeation models such as the one shown in Figure 9 are, therefore, useful to enable the simultaneous study of intestinal digestion and drug absorption, with the aim of increasing predictability of in vitro tools. The device generally consists of two compartments: one for digestion and one for absorption, separated by a membrane, such as a monolayer of MDCK (Madin–Darby Canine Kidney cells), Caco-2 cells [83], or a synthetic membrane [84]. It is also possible to perform a standard lipolysis test as described above, then place the samples taken from the digestion vessel on Franz cells [85]. As Caco-2 cells are not always compatible with LBFs or pancreatin for lipolysis of lipids [85], it is sometimes preferable to use a synthetic membrane such as PermeaPad^®^ [86] or a piece of rat intestinal tissue as an ex vivo permeation setup [68]. In this type of ex vivo test, a fragment of intestine can be used to represent the absorption part of the combined model [87]. Finally, in situ absorption is an alternative to ex vivo systems; an in vitro model of lipid digestion is coupled directly to in situ intestinal perfusion in an anesthetized rat. The contents of the digestion vessel are transferred to the animal’s jejunum using a peristaltic pump [88]. However, these types of systems are less ethical than the use of cells or synthetic membranes.

In vitro–in vivo correlations on lipid formulations have been investigated with this type of combined system. In 2021, Klitgaard and colleagues developed a level D correlation between in vitro and in vivo rat data for cinnarizine [85]. The same level of correlation was obtained for carvedilol, with in vivo data on a dog model [89]. In both cases, the order of performance of lipid formulations in vivo was correlated with data generated by a lipolysis–permeation model. Recently, Sirvi and colleagues have been working on nilotinib, and more specifically on the complexation of this molecule, or the formation of a salt. By comparing results with in vivo data, the authors succeeded in obtaining a correlation between in vitro and in vivo data [90]. Finally, Mageshvaran et al. investigate the inhibition of dasatinib precipitation in a SNEDDS formulation and develop IVIVC using an in vitro lipolysis–permeation model [91].

Without linking in vivo data with in vitro data, this model is also useful for the formulation design of LBFs. Alvebratt and colleagues used the μFLUX™ equipment marketed by Pion to adapt a combined lipolysis–permeation model [92]. This type of installation uses fiber optics to calculate the concentration of active molecules in situ using a UV spectrophotometer. Other studies published in the literature show the relevance of the results obtained with this type of model on different active ingredients: ritonavir [84], danazol [93], or fenofibrate [93].

The notion of coupling two models is important because the data obtained during lipolysis and during permeation are linked and indispensable for establishing a strong correlation. Crum et al. were unable to correlate in vitro data using a pH-stat lipolysis model with in vivo data, but by combining in vitro lipolysis with in situ perfusion in an anesthetized rat, they were able to predict the in vivo performance observed in previously published studies [88]. Similarly, a study carried out on danazol and fenofibrate highlighted the lack of predictability of the data generated by lipolysis, but this lack of predictability was compensated by adding the permeation data generated during the experiment, which enabled the creation of a correlation with in vivo data [83]. This study also highlighted the in vitro behavior of LBFs, which differ according to the type of formulation chosen, with reference to LFCS [5]. Finally, work carried out on cinnarizine with this type of coupling also demonstrated the impact of lipolysis–permeation coupling compared with lipolysis alone on the prediction of in vivo performance [86].

The combined lipolysis–permeation model is, therefore, relevant to the study of lipid formulations. Absorption is an important phenomenon to take into account when predicting in vivo bioavailability. Despite some lack of predictability in certain situations, such as the use of an ex vivo absorption model [68], this coupling remains a useful method for developing a lipid formulation.

##### TNO TIM-1 and Permeation

This TNO TIM-1 combined with the Caco-2 cell model is suitable for predicting the bioavailability of a drug. It enables researchers to assess how the formulation will interact with the gastrointestinal tract and intestinal barrier, ensuring efficient drug release, digestion, and absorption. In this combined model, digested samples from the aforementioned TIM-1 system are added to the Caco-2 cell monolayer after ultracentrifugation, filtration, and dilution, with the aim of studying the absorption of the sample resulting from dynamic digestion [94].

This method was first developed in 2009 for lycopene and α-tocopherol. In the dynamic digestion study, α-tocopherol was stable during digestion. In contrast, lycopene release was significantly lower, with a loss of around 25% of the initial lycopene. In Caco-2 cells, the saturable uptake of α-tocopherol and lycopene and their bidirectional passage through the monolayer pointed to protein-mediated uptake. Permeability tests at different concentrations for pure and formulated molecules showed that the percentages of pure compounds absorbed were significantly higher when the lowest concentrations were used. By combining the TIM system with Caco-2 cells, the authors were able to highlight a lower bioavailability for lycopene than for α-tocopherol. Based on the literature and their laboratory tests, the authors concluded that lycopene is poorly absorbed compared to α-tocopherol during digestion in the gastrointestinal tract [94].

Recent advances underline the growing interest in combined in vitro systems. A recently published patent describes a dynamic platform that continuously couples dissolution or digestion with cell-based permeability assays. This approach enables a more direct and physiologically relevant evaluation of drug solubilization and absorption, highlighting the potential of such combined models for lipid-based formulations [95].

### 4.2. In Vivo Data

In vivo data play a crucial role in establishing in vitro–in vivo correlations for lipid-based formulations. These data can be sourced from clinical trials on humans during the drug development process, where pharmacokinetic profiles are generated to evaluate the drug’s absorption, distribution, metabolism, and excretion. Such studies provide high-quality data directly relevant to the target population and are particularly valuable for building level A IVIVC models, which offer detailed predictive insights. Alternatively, preclinical biopharmaceutical studies using animal models, such as rats, dogs, or pigs, are commonly employed to generate in vivo data. These models are particularly useful in the early stages of development when human data may not yet be available.

Since 1959, animal experimentation has been tightly controlled and based on an ethical approach known as the 3Rs rule [96]: “Reduce” the number of animals used in studies and ensure that studies are reduced to those considered essential for the purpose, “Replace”, if possible, the animal with in vitro tests or computer modeling, and “Refine” animal experimentation to ensure animal welfare. However, it is important to keep in mind that no model is perfect; what is important is to know the physiological characteristics of the model in order to be able to interpret the results in the best possible way and to be aware of the limits of the study model. For example, unlike humans, rats do not have any gall bladder and release bile salts without interruption. The concentration of bile salts is higher in rats than in humans. Also, a dog’s stomach is not in the same position as a human’s. This is due to its mode of movement. The gastric emptying could then be affected.

Musther et al. have evaluated the predictivity of results obtained in animals compared with those observed in humans [97]. This study concludes that there are not strong and predictive linear correlations between overall and single-species animal drug bioavailability and human values. Data interpretations must be based on an understanding of physiological, metabolic, and transporter-related information that affects bioavailability on a species-by-species basis. Extrapolations between animals and humans must be carried out rigorously, taking interspecies variability into account. In the case of lipid-based formulations, which involve specific mechanisms of digestion and absorption, it is particularly critical to account for the physiological and mechanistic differences inherent to each animal model.

## 5. In Silico Models for Predicting In Vivo Performance

PBPK (Physiologically Based PharmacoKinetic) and PBBM (Physiologically Based Biopharmaceutics Modeling) in silico models are increasingly used in pharmaceutical development [98]. According to a review published in 2024, from 2019 to 2023, out of 243 novel drugs approved by the FDA, 74 included PBPK modeling [99]. Since 2013, there has been a clear increase in the use of in silico models to support regulatory decisions for new pharmaceutical substances, supported by regulatory authorities. This increase reflects the growing interest of the pharmaceutical industry in these predictive models. Beyond their regulatory value, in silico models are also of great interest to the pharmaceutical industry because they align with the 3Rs principle and corporate social responsibility (CSR) by reducing both animal experimentation and the amount of laboratory work required. Among the most widely used tools are GastroPlus^®^, Simcyp^®^, and PK-Sim^®^. However, this innovative approach is still not being used to its full potential. Future advancements in these platforms are expected to enhance their predictive capabilities. It is also important to note that in vitro data may not always accurately reflect in vivo conditions. Therefore, a solid understanding of each input parameter and its impact on model predictions is essential to ensure the credibility and reliability of PBPK modeling. PBPK models can be used to predict the in vivo behavior of an active substance or formulation based on biopharmaceutical data generated in vitro (PBBM models) or on the physiology and pharmacokinetics of the subject (PBPK models). However, these models are not always suitable for lipid formulations [100]. Indeed, the phenomena mentioned above, such as lipid digestion, are not always taken into account in models. These phenomena are important, however, as they can modify the biopharmaceutical phase of the product and hence its in vivo behavior.

### 5.1. GastroPlus^®^

**Figure 10:**
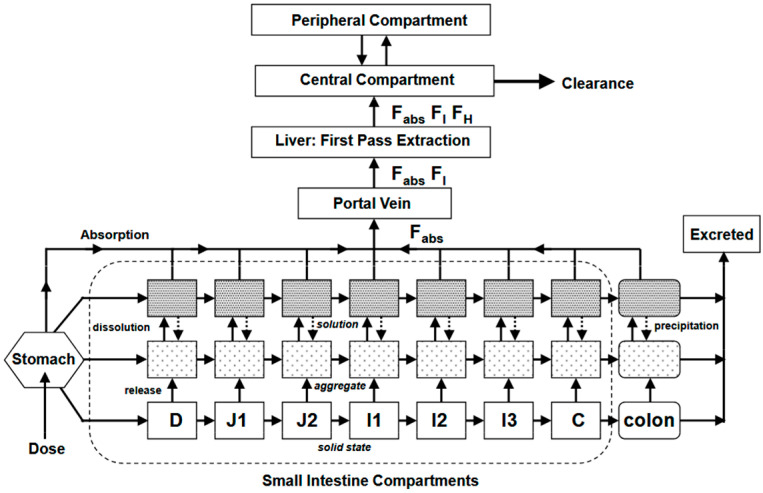
Schematic of Advanced Compartmental Absorption Transit (ACAT™) model (from Chow et al., 2013 [103]).

GastroPlus^®^ (Simulations Plus, Inc., Lancaster, CA, USA) is an advanced simulation software package developed by Simulations Plus, designed to model drug absorption by various routes, such as intravenous, oral, buccal, ocular, inhalation, cutaneous, subcutaneous, and intramuscular. It is the second most widely used software for developing PBPK models, according to a 2024 study of new drugs approved by the FDA over the last five years [99]. GastroPlus^®^ is based on the mechanistic Advanced Compartmental Absorption Transit (ACAT™) model (see Figure 10), which simulates the absorption of oral formulations from the gastrointestinal tract [101]. GastroPlus^®^ also incorporates useful modules for building pharmacokinetic models, such as ADMET Predictor^®^ for predicting input parameters from the molecular structure of the molecule, PBPK Plus™ and PEAR™ for enriching simulation capabilities by taking into account factors such as the weight, volume, and blood perfusion rates of human organs, and DDI for studying drug interactions.

GastroPlus^®^ also has its own module for developing in vitro–in vivo correlations. The IVIVCPlus™ module is an optional add-on that offers a convenient way to develop correlations between in vitro and in vivo release [102].

GastroPlus^®^ software was used for studies on lipid-based formulations. An IVIVC was developed on SLN-type (solid lipid nanoparticle) lipid formulations using the IVIVCPlus™ module using the Loo–Riegelman law [104]. This study concluded that SLNs could be a potential drug carrier for furosemide with improved bioavailability upon oral administration. The in vivo performance of ketoconazole SLNs in rats was also predicted with GastroPlus^®^ [105]. The tool was useful in guiding the choice of excipients used in SLNs. O’Shea and colleagues used the software to study the effect of feeding on a solid lipid dispersion versus the marketed micronized formulation of fenofibrate in mini-pigs. The model developed accurately predicted the fasting plasma profile of lipid dispersions and marketed micronized formulations [106]. In addition, PBPK modeling on adsorption-solidified rifampicin SNEDDS revealed a good correlation between the in vitro release profile and in vivo data and validated the advantages of using a solidified SNEDDS [107]. Ćetković et al. used in silico PBPK modeling to select an appropriate delivery strategy for simvastatin SMEDDS systems [108]. SMEDDS were mixed with polymethacrylate polymers to form a gel that releases the active substance at pH values above 7, thus preventing degradation of the molecule. The default precipitation available in the software was used for the simulations. To simulate the specific effect of SMEDDS gel formulations, the drug dissolution profiles observed in vitro were used as input data for the simulations [108]. Recently GastroPlus^®^ assisted in the development of controlled-release Viagra^®^ (Pfizer, New York, NY, USA) containing Poloxamer-188 [109]. In silico evaluation using the software predicted significant variation in Cmax, tmax, t1/2, and AUC between formulations. The combination of vitro and silico in pharmaceutical development is important and has been demonstrated with GastroPlus^®^ on the BCS 4 albendazole molecule [110].

Many studies have applied GastroPlus^®^ to model lipid-based formulations, primarily focusing on solidified Self-Emulsifying Drug Delivery Systems (SEDDSs) adsorbed onto carriers or Nanostructured Lipid Carriers (NLCs). However, liquid SEDDSs, which have distinct digestion and absorption dynamics, remain not sufficiently explored. Investigating the bioavailability of liquid SEDDS using GastroPlus^®^ could reveal the software’s ability to predict in vivo performance based on formulation variations. This approach would also help identify any limitations in modeling the specific mechanisms of liquid LBFs.

### 5.2. Simcyp^®^

**Figure 11:**
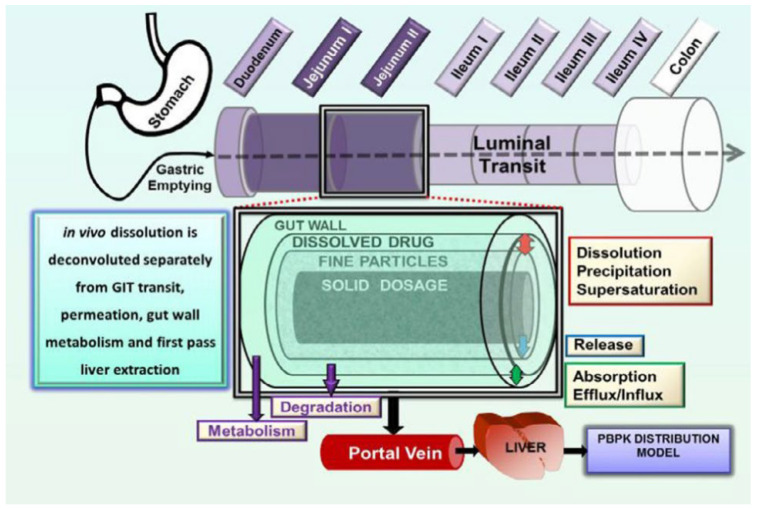
Schematic of Advanced Dissolution Absorption and Metabolism (ADAM) model (from Kostewicz et al., 2014 [111]).

Simcyp^®^ is a human population-based simulator that provides pharmacokinetic and pharmacodynamic models. It is currently the most widely used software for new drugs approved by the FDA over the last five years [99]. This software uses the ADAM (Advanced Dissolution Absorption and Metabolism) model (see Figure 11), a multi-compartment model of gastrointestinal transit fully integrated into the simulator for humans, rats, mice, and dogs [111]. The ADAM model identifies the gastrointestinal tract as a stomach, seven sections of small intestine, and a colon compartment, in each of which the drug can exist in several states simultaneously: unreleased, undissolved, dissolved, or degraded [111,112].

Posaconazole, class BCS 2, has been studied with in silico prediction models such as SimCyp^®^ [113]. This study demonstrates the predictive power of PBPK modeling to assess the impact of the pH of a formulation for two posaconazole suspensions on systemic exposure. Simulation results were compared with clinical data and well matched in terms of gastric dissolution, intestinal supersaturation, and the solid amount of active ingredient present in the upper small intestine. Howgate and colleagues used SimCyp^®^ to predict median in vivo clearances from in vitro data. On the basis of in vitro metabolism data and in vivo human clearance values collected for 15 drugs from the literature, they showed that predicted median clearance values did not exceed twice the values observed for 93% of orally administered drugs [114]. SimCyp^®^ has also been successfully applied to the development and validation of IVIVC for controlled-release formulations of the BCS 1 drug metoprolol [115]. This study also led to the design of a new once-daily formulation by optimizing the dose to obtain the desired in vivo profile. The established IVIVC was validated to obtain the required in vitro dissolution profile from the estimated in vivo dissolution profile. This in silico pharmacokinetic prediction model is also used to characterize drug–drug interactions [116,117,118]; for example, the simulator demonstrated that 70% of erlotinib metabolism was mediated by CYP3A4, with the remainder eliminated by CYP1A2. On this basis, the impact of co-administration of ketoconazole (a CYP3A inhibitor) was simulated, and the twofold increase in erlotinib exposure was found to be consistent with the results of a clinical study [119]. Finally, this software is useful for anticipating the food effect and comparing fasted and fed administrations, as in the example of ritonavir published in 2020 [119].

Despite all the studies published in the literature on this model, there are few studies of lipid-based formulations using this software. Its use is effective for most of the molecules tested, but the complex case of LBF administration may raise questions about the reliability of the results generated.

### 5.3. gPROMS Formulated Products^®^

Based on the gPROMS^®^ advanced process modeling platform from Siemens and Process Systems Enterprise, gPROMS Formulated Products^®^ (PSE, London, UK) offers capabilities for the food and agrochemical industries, enabling model-based design and optimization of process operations such as reaction, crystallization, wet and dry milling, spray drying, wet and dry granulation, blending, and tableting [120]. gPROMS Formulated Products^®^ uses the mechanistic absorption model built using gCOAS (Computational Oral Absorption Simulation Framework) based on the gPROMS^®^ language. This mechanistic model integrates physicochemical and formulation characterization information to simulate the dissolution of an orally administered tablet in the gastrointestinal tract and the absorption of drugs from the tract while taking physiology into account. This tool can be used to understand the impact of drug solubility on dissolution and absorption in vivo.

A model using gCOAS was used to understand the impact of drug solubility on dissolution and absorption in vivo, with solubility modified by the addition of SLS (Sodium Lauryl Sulfate) [121]. The mechanistic model integrates molecule and formulation characteristic information to simulate the dissolution of an orally administered tablet in the gastrointestinal tract (GIT) and the resulting absorption. With the pharmaceutical compounds renamed X and Y, the model indicated that due to the high solubility of compound X in the GIT, the presence of SLS only slightly increased the dissolution rate, with no impact on absorption. For compound Y, the model predicts a significant impact of SLS concentration on drug release [121]. Recently, Kesharwani and colleagues have highlighted the value of using the software “Global System Analysis” (GSA) in pharmaceutical development. In this study, this tool was used to provide insight into the active substance, the final product, or the physiological parameter that would have an impact on the Cmax and AUC of dipyridamole. Mechanistic modeling studies have shown that a precipitation-integrated modeling approach can adequately predict mean plasma profiles, Cmax, and AUC of in vivo clinical study results [122].

Although no articles on LBFs were found during the literature search carried out for this article, this tool seems to be a good opportunity for future studies. gPROMS Formulated Products^®^ also seems to be interesting software for its industrial advantage of linking modules, enabling a complete simulation of the manufacturing process right through to intestinal absorption.

### 5.4. PK-Sim^®^

**Figure 12:**
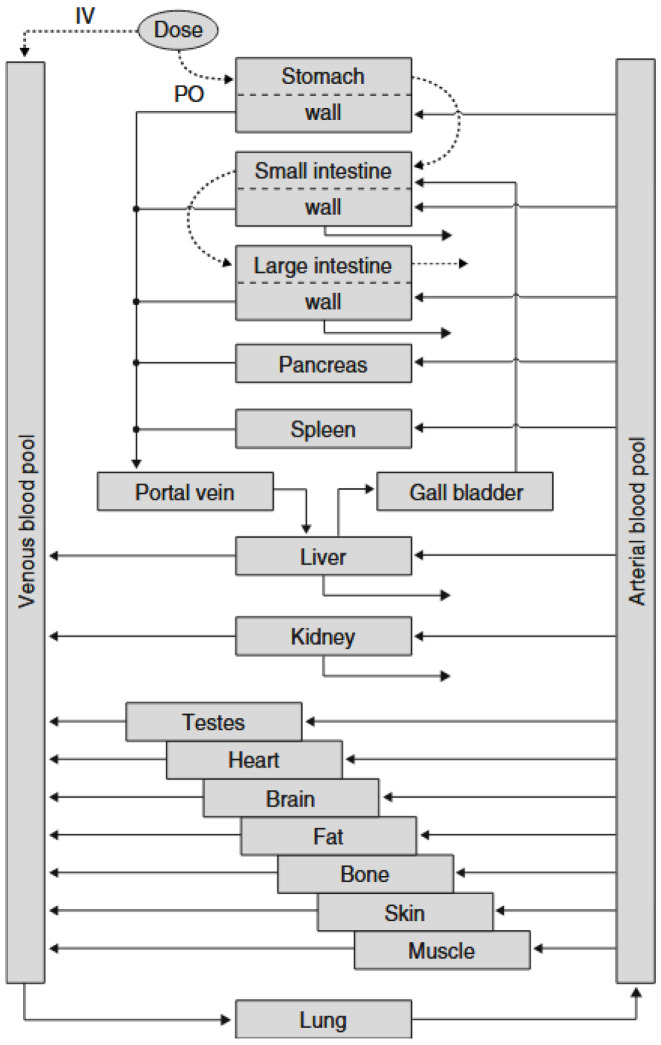
Schematic of the structure of the PK-Sim^®^ model (from Dressman et al., 2008 [123]). The dashed arrows represent the transit of the active molecule depending on the administration routes. The solid arrows represent the connections between the modules.

PK-Sim^®^ is a software tool for pharmacokinetic modeling based on body physiology. It is distributed by Bayer Technology Services GmbH (Leverkusen, Germany). It provides rapid access to all anatomical and physiological parameters relevant to humans and the most common preclinical animal models (mouse, rat, mini-pig, dog, and monkey), which are contained in the integrated database [111]. Unlike most PBPK modeling tools, PK-Sim^®^ offers different model structures, for example, to take account of the important differences between small and large molecules. The structure of the gastrointestinal tract model consists of several segments (stomach, duodenum, upper and lower jejunum and ileum, respectively, cecum, ascending, transverse, descending, and sigmoid colon, and rectum) [111]. As shown in Figure 12, in PK-Sim^®^, all organs are presented as compartments, and each compartment is subdivided into a vascular space and an extravascular space. The vascular space is further subdivided into plasma and red blood cells, while the extravascular space is subdivided into interstitial and cellular space.

PK-Sim^®^ software was evaluated in a review of three cases of pharmaceutical molecules [124]. Firstly, cilostazol (BCS 2) was chosen as the model drug due to the availability of a detailed experimental data set. The decrease in absorption with increasing particle size was well predicted by the model. In this study, the impact of three different oral formulations and feeding conditions on the pharmacokinetics of a substance named X (BCS 2) was also predicted using this software. The third example described the use of the software to support the development of a molecule called Y in a combined immediate-release (IR) and controlled-release (CR) formulation. Unfortunately, no in vitro–in vivo correlation could be established on the basis of the data. With the help of a PBPK model, information on the impact of changes in the composition of a combined IR-CR formulation was generated, and the optimal composition in terms of immediate release and controlled release of the solid dose was proposed.

PK-Sim^®^ can be used to predict the in vivo performance of a pharmaceutical product in animals; indeed, oral absorption of drugs from cilostazol suspensions has been quantitatively predicted in dogs [125]. In rats, a continuous absorption model has been developed to simulate drug absorption in the gastrointestinal tract [126]. Finally, the physiological model of gastrointestinal transit and absorption developed for rats and humans has been extended for use in monkeys [127]. The model can then be used to predict the fraction of dose absorbed passively after oral administration and to assess the influence of inter-individual physiological variability on oral absorption [127].

Nifedipine is a BCS 2 active substance marketed as a softgel capsule containing glycerol, purified water, saccharin, peppermint oil, and macrogol 400 [128]. After several in vitro dissolution studies, Thelen and colleagues built a PBPK model using PK-Sim^®^ software [128]. At doses of 5 and 10 mg, the plasma concentration–time profiles of orally administered nifedipine were well described by the PBPK model. In contrast, concentration profiles at the 20 mg dose could only be correctly described on the assumption of precipitation [128].

Data generated in silico with PK-Sim^®^ on lipid-based formulations are insufficient, as is the case with SimCyp^®^ and gPROMS Formulated Products^®^. For these three in silico models, the small number of studies on LBFs may call into question the reliability of these models for the study of LBFs where lipolysis impacts bioavailability.

## 6. Proposed Strategy for IVIVC in LBFs

In traditional IVIVC development, when the dissolution is the limiting factor, the process involves directly linking in vitro dissolution profiles with observed in vivo pharmacokinetic curves through simple linear regression. However, this approach might not be sufficient for lipid-based formulations, where the release of the active ingredient could be influenced by many complex factors beyond dissolution alone.

**Figure 13:**
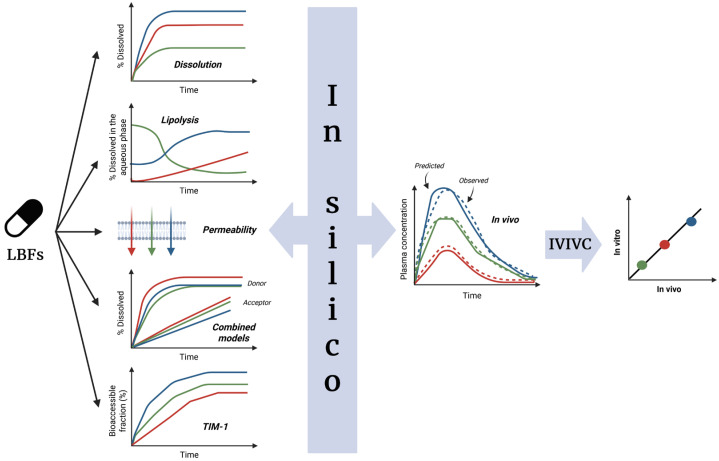
Proposed strategy for IVIVC in LBFs. The red, blue, and green colors represent different LBFs with distinct in vitro and in vivo performances.

As shown in the proposed scheme (Figure 13), multiple in vitro measurements such as lipolysis profiles, dissolution data, apparent permeability (Papp), combined models, and dynamic gastrointestinal simulations like TIM-1 could first be collected. These diverse data would reflect important processes, including lipid digestion, solubilization, and intestinal permeability. These in vitro results could then be integrated into an in silico model that would simulate the drug’s behavior in the gastrointestinal tract. This model might predict pharmacokinetic profiles by accounting for digestion, micelle formation, permeability, and other mechanisms specific to LBFs. The predicted PK profiles could then be compared with observed in vivo data to establish a more reliable IVIVC.

This multi-step approach suggests that measuring dissolution alone might not be enough to link in vitro and in vivo performance for lipid-based formulations. Instead, combining experimental data with mechanistic modeling, as illustrated in the scheme, could offer a better understanding and prediction of how these complex formulations behave in the body.

## 7. Conclusions

To improve the development of IVIVCs for LBFs, it is essential to adopt a global approach integrating in vitro, in vivo, and in silico methodologies. Future efforts and prospective studies should focus on defining and studying the predictability of all in vitro tests, on one hand to enable more accurate translation of in vitro results into in vivo performance and thus develop IVIVCs, and on the other hand to identify relevant information for the construction of in silico models in order to effectively predict the in vivo performance of lipid-based formulations. To fill the current gaps in in silico modeling of lipid-based formulations, it is necessary to adapt both the input data and the model equations to account for the complexity of these formulations. Improving in silico platforms to take better account of lipid digestion, permeation enhancement, micelle formation, or lymphatic transport will, therefore, pave the way for more reliable predictions.

## Data Availability

No new data were created or analyzed in this study. Data sharing is not applicable to this article.

## Abbreviations

The following abbreviations are used in this manuscript:

SEDDS	Self-Emulsifying Drug Delivery System
SMEDDS	Self-Microemulsifying Drug Delivery System
SNEDDS	Self-Nanoemulsifying Drug Delivery System
LFCS	Lipid-Based Formulation Classification System
HLB	Hydrophilic–Lipophilic Balance
USP	United States Pharmacopeia
API	Active Pharmaceutical Ingredient
AUC	Area Under Curve
CMC	Critical Micellar Concentration
FSM	Fed Stomach Model
DGM	Dynamic Gastric Model
PBPK	Physiologically Based PharmacoKinetic
MDCK	Madin–Darby Canine Kidney
PBBM	Physiologically Based Biopharmaceutics Modeling
NLCs	Nanostructured Lipid Carriers
ACAT	Advanced Compartmental and Transit
ADAM	Advanced Dissolution Absorption and Metabolism
COAS	Computational Oral Absorption Simulation
GIT	Gastrointestinal tract
GSA	Global System Analysis
CR	Controlled Release
IR	Immediate Release
Papp	Apparent Permeability
CSR	Corporate Social Responsibility
SLNs	Solid Lipid Nanoparticles
